# SOP: treatment of delirium

**DOI:** 10.1186/s42466-021-00110-7

**Published:** 2021-03-04

**Authors:** Juraj Kukolja, Jens Kuhn

**Affiliations:** 1 Department of Neurology and Clinical Neurophysiology, Helios University Hospital Wuppertal, Heusnerstr. 40, 42283 Wuppertal, Germany; 2 Faculty of Health, Witten/Herdecke University, 58448 Witten, Germany; 3 Medical Faculty, University of Cologne, 50937 Cologne, Germany; 4 Department of Psychiatry and Psychotherapy, Medical Faculty, University of Cologne, Cologne, Germany; 5 Department of Psychiatry, Psychotherapy and Psychosomatic Medicine, Johanniter Hospital Oberhausen, EVKLN, Steinbrinkstraße 96a, 46145 Oberhausen, Germany

## Abstract

**Introduction:**

Delirium is a frequent complication in hospitalised patients, often leading to difficulties in patient management and is associated with increased morbidity and mortality. Most patients in intensive care units develop delirium, however, it is also frequently observed in non-intensive care unit settings. Risk factors are, among others, older age, brain pathology, severe trauma, orthopaedic or heart surgery, metabolic or electrolyte dysregulations, infections and polypharmacy. The most important measures to prevent and treat delirium are recognition and removal of risk factors and causes. Although delirium is a very common and serious complication, evidence for pharmacological treatment is poor, and guidelines remain controversial. Accordingly, non-pharmacological treatments have gained increasing attention and should be applied. Based on current literature, guidelines and personal recommendations, we developed a standard operating procedure (SOP) encompassing non-pharmacological and pharmacological treatment of delirium.

**Comments:**

In order to prevent delirium, risk factors should be identified and taken into account when planning the hospital stay and treatment. Prevention should include multimodal non-pharmacological interventions. The treatment of delirium should encompass the elimination of potential causes and non-pharmacological interventions. Pharmacological treatment should be used in a time-limited manner and in the lowest possible dose for the management of highly stressful symptoms or high-risk behaviour.

**Conclusion:**

The SOP provides a pragmatic algorithm for the non-pharmacological and pharmacological treatment of delirium.

**Supplementary Information:**

The online version contains supplementary material available at 10.1186/s42466-021-00110-7.

## Introduction

Delirium is the most frequent psychiatric complication in hospitalised patients, associated with considerable difficulties in patient management, prolonged hospital stay, worse clinical outcome, increased mortality, higher rate of cognitive decline and institutionalisation [1].

Reports of delirium prevalence on general wards vary between 11 to 42%, with a prevalence of approximately 50% in patients aged more than 65 years and up to 80–89% in an intensive care setting [1].

Commonly, delirium encompasses disorientation, disorganised thinking, delusions, visual or acoustic hallucinations, and vegetative symptoms. Fluctuations of symptom severity with higher incidence during the night are typical. The most frequent hyperactive delirium is characterised by psychomotor agitation and restlessness. The less frequent hypoactive delirium, sometimes challenging to diagnose, presents with reduced psychomotor activity and level of arousal especially in the elderly. Mixed (hyper-hypoactive) forms can also be observed. Usually, patients with delirium present memory disorders and have amnesia for the affected period of time.

Predisposing factors for a delirium are older age, cognitive impairment (25–50% of delirium patients suffer from pre-existing dementia), multiple comorbidities, and treatment with multiple (> 6) medications.

In spite of its high prevalence in hospitals and its impact on the clinical course, evidence for effective treatment options remains scarce. A limited number of studies supports the use of pharmaceutical interventions [1–3]. In recent years, therefore, non-pharmaceutical interventions have gained increasing importance.

The present standard operating procedure (SOP) for prevention and management of delirium is based on current literature, but the recommendations for pharmacological treatment are based on limited evidence.

## Definition

According to the DSM-5 (Table S1 [4];), delirium is defined as a disturbance in attention and awareness, associated with cognitive impairment in the domains of memory, orientation, language, visual-spatial skills, or perception. Usually, the onset is acute or subacute and the severity tends to fluctuate. The symptoms may not be explained by a known neurocognitive disorder or severely reduced level of arousal. Additionally, there should be evidence that the symptoms are the result of a clinical condition such as the presence of an acute disease, intoxication or withdrawal.

## Comments

### Diagnostic procedures

**Fig. 1:**
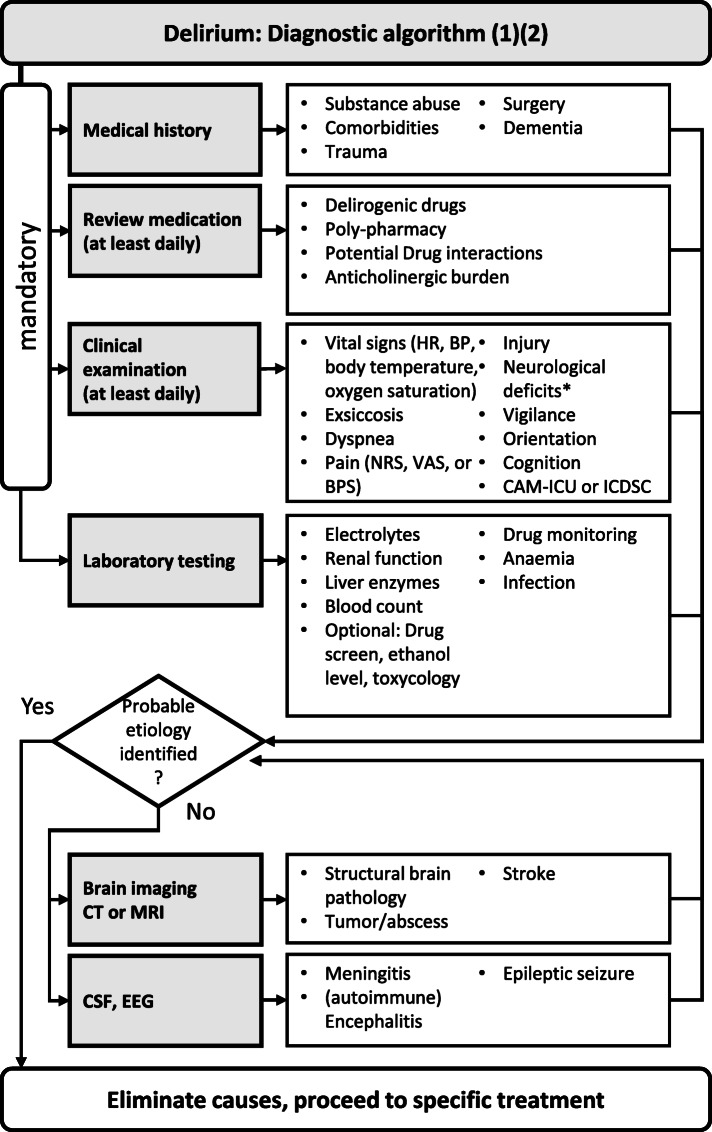
Flow Chart for the diagnosis of delirium. BPS: behavioral pain scale, CAM-ICU: Confusion Assessment Method in the Intensive Care Unit, CT: computed tomography, CSF: cerebrospinal fluid, EEG: electroencephalography, FORTA: Fit for the Aged list, ICDSC: Intensive Care Delirium Screening Checklist, MRI: magnetic resonance imaging, NRS: numeric rating scale, VAS: visual analogue scale

In order to detect a delirium, disturbances in orientation, concentration, vigilance and attention need to be identified. A useful tool for the detection and quantification of a delirium are the CAM-ICU (Confusion Assessment Method in the Intensive Care Unit, http://www.icudelirium.org, Table S3) and the ICDSC (Intensive Care Delirium Screening Checklist, [5], Table S4). Since delirium is unspecific with respect to its aetiology, but elimination of potential causes is of primary importance, medical history and body examination have to be conducted with great care. We propose a pragmatic procedure in the search for delirium aetiology in Fig. 1.

The medical history should encompass a potentially causal event (e.g., trauma or surgery), pre-existing medical conditions or cognitive disorders, medication, substance abuse. For older patients, the PRISCUS list [6] or the FORTA list (Fit for the Aged, https://www.umm.uni-heidelberg.de/klinische-pharmakologie/forschung/forta-projekt/; also available as a mobile device app, [7]) can be used to identify unfavourable medication. Clinical examination should focus on potential neurological signs and deficits, lung auscultation (pneumonia), abdominal palpation and auscultation, consistency of the skin (dehydration), presence of oedema or elevated body temperature. Furthermore, pain should be assessed once per shift, ideally using rating scales such as the visual analogue scale (VAS), numeric rating scale (NRS) or the behavioral pain scale (BPS) where applicable. Routine laboratory should be assessed to detect inflammation or infection, electrolyte or metabolic dysregulations. If routine laboratory findings do not deliver sufficiently conclusive results, additional diagnostic procedures such as X-ray of the lungs, cranial computed tomography (CT) or magnetic resonance imaging (MRI), or lumbar puncture should be considered. These are also necessary to identify different treatable neurocognitive disorders.

### Common causes

Causes of delirium can be manifold and may involve several simultaneous pathologies simultaneously. The underlying mechanisms of cerebral dysfunction leading to delirium are not fully understood. It is thought that as a common final pathway an overactivity of the dopaminergic system and an underactivity of the cholinergic system which are reciprocally interconnected. Depending on the underlying disease, other factors may contribute to the development or maintenance of delirium, such as inflammatory mediators (cytokines, e.g., interleukin 1 and 6), electrolyte or metabolic dysregulations, vascular pathology, traumatic brain injuries, drug interactions or psychiatric diseases (see Table 2). Delirium is more frequent after change of environment (e.g. admission to a hospital) and during nighttime.

### Non-pharmacological prevention and treatment of delirium

**Fig. 2:**
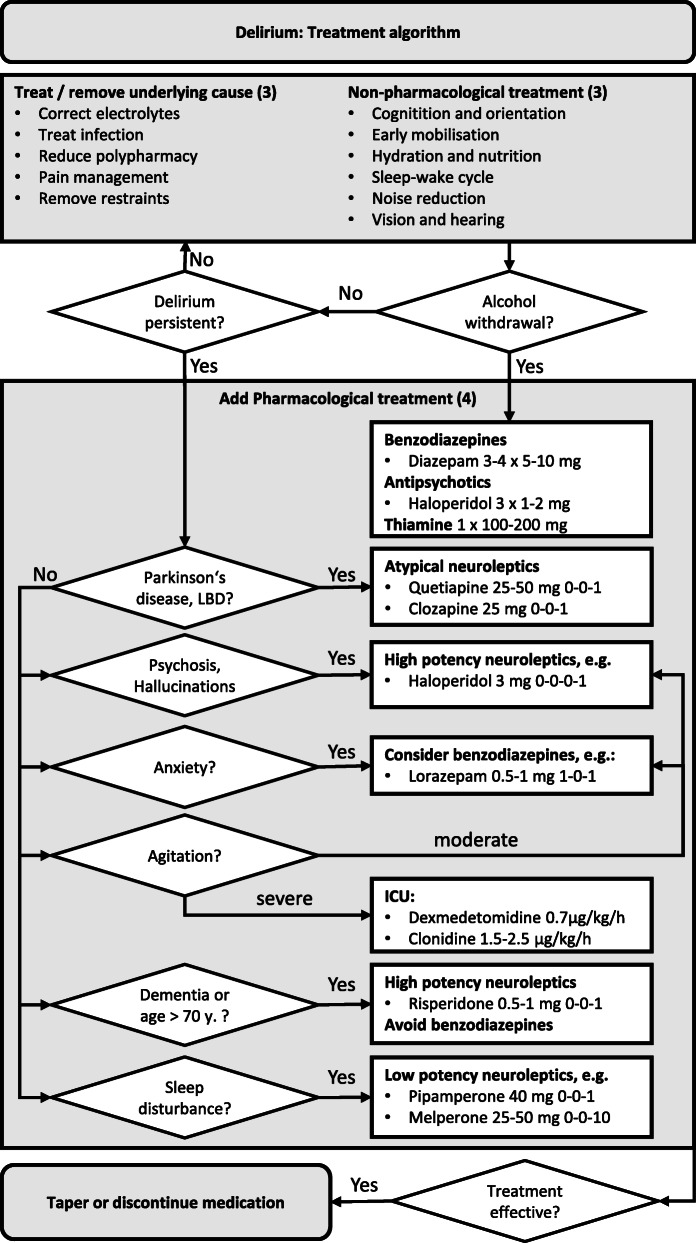
Flow Chart for the therapy of delirium. ICU: intensive care unit. *Look for neurological signs and deficits including: meningism, paresis, pyramidal signs, sensory deficits, vision loss or deficits, ataxia, apraxia, nystagmus, cranial nerve deficits etc.

In order to prevent delirium, avoidance and elimination of causes and risk factors have to be prioritised (Table S2, Fig. 2). Furthermore, multicomponent non-pharmacological interventions have proven effective in the prevention of delirium (Table 1, Fig. 2, [8]).

**Table 1:** Multi-component, non-pharmagological interventions to prevent and treat delirium

Cognition and orientation	Orienting communication
	Cognitive stimulation
	Avoidance of sensory deprivation
	Daily schedule, name plates of staff
Early Mobilisation	Ambulation and exercises
	Avoidance of immobilisation
Hydration and Nutrition	Sufficient fluid intake or fluid supplementation
	Sufficient nutrition, feeding assistance
Pain management	Screen for pain symptoms (1 x per shift, VAS, NRS, or BPS)
	Adequate pain treatment
Medication	Check medication daily
	Reduce and eliminate unnecessary medication, especially if an anticholinergic mechanism is part of the pharmacodynamic profile (see also: PRISCUS list [6], FORTA list [7], https://americandeliriumsociety.org/files/ACB_Handout_Version_03-09-10.pdf)
Vision and hearing	Hearing and vision aids
Sleep-wake cycle	Noise reduction at night (schedule adjustment etc.)
	Activity during daytime

In the intensive care unit (ICU) setting, adherence to interventions including early breathing trials, restrictive use of analgesia and sedation, delirium assessments on a regular basis, early mobilisation and exercise, and family engagement were associated with decreased rates of death, delirium and coma [9].

Although the evidence for non-pharmacological interventions is more robust for the prevention than for the treatment of delirium [1], the authors of this SOP encourage the use of non-pharmacological interventions (Table 1, Fig. 2) also when delirium is present since they will not inflict harm and may at least help individual patients [10, 11].

### Pharmacological treatment

Delirium, especially if hyperactive and associated with agitation and aggression, can result in dangerous situations for patients and health care professionals. Antipsychotics are commonly used as first-line medication in order to confront these situations, although the evidence for their use to treat delirium in non-ICU or ICU settings is limited [1, 2]. While some high quality studies are in support of a pragmatic use of antipsychotics with or without benzodiazepines for delirium [2, 3], a Cochrane database meta-analysis from 2018 did not find sufficient data to support or refuse the use of typical or atypical antipsychotics for the treatment of delirium [12]. Furthermore, neither haloperidol nor ziprasidone were effective in shortening the duration of delirium or coma in critically ill patients [13]. A routine recommendation is therefore not possible.

Antipsychotic treatment should be limited to the management of symptoms imposing a safety risk for the patient or medical staff or which are highly stressful for the patient (such as hallucinations, agitation, aggression). Based on the mechanism of action, high potency neuroleptics (such as haloperidol or risperidone as an alternative) should be used for psychotic symptoms or hallucinations (Table 2, Fig. 2) [10, 11]. When compared to the treatment of schizophrenia, a significantly lower dose (i.e., 3 mg haloperidol equivalents) has been shown to be more favourable in terms of overall outcome. Low potency neuroleptics with sedative effects (such as melperone or pipamperone) and lorazepam are useful for the treatment of agitation or an inverse sleep-wake-cycle (Table 2, Fig. 2).

**Table 2:** Selected drugs for the treatment of delirium (in alphabetical order per class)

drug	dose		antagonism	anti-psychotic	sedating	dementia	parkinson’s	caveat
**Neuroleptic drugs**								
clozapine	25 mg	0–0-1	5-HT_2A,_ 5-HT_2C_, α_1_	**+**	**+**	**+**	**+**	Risk of agranulocytosis
haloperidol	1 mg	1–0-1	D_2_	**+++**	**–**	**–**	**–**	no i.v. application
melperone	25–50 mg	(1)-0–1	D_2_, α_1_	**+**	**++**	**–**	**–**	Dose depending on agitation level, available in several EU countries
pipamperone	40 mg	(1)-0–1	5-HT_2A_, D_4_, α_1_	**+**	**++**	**+**	**–**	Dose depending on agitation level
quetiapine	25–50 mg	(1)-0–1	5-HT_2A_, D_2_	**+**	**++**	**+**	**+**	Dose depending on agitation level
risperidone	0,5–1 mg	1–0-1	D_2_, 5-HT_2A_	**+++**	**–**	**+**	**–**	Dose reduction in renal insufficiency
**α**_**2**_**-Agonists**								
clonidine	1.5–2.5 μg/kg/h		α_2_ (agonist)		**+++**	**+**	**+**	ICU, bradycardia
dexmedetomidine	0.7 μg/kg/h		α_2_ (agonist)	**–**	**+++**	**+**	**+**	ICU, bradycardia, contraindication stroke
**Benzodiazepines**								
diazepam	5 mg	prn	GABA_A_	**–**	**+++**	**–**	**–**	short term
lorazepam	0.5–1 mg	prn	GABA	**–**	**+++**	**–**	**–**	short term
midazolam	7.5–15 mg	prn	GABA_A_	**–**	**+++**	**–**	**–**	short term
oxazepam	10 mg	prn	GABA_A_	**−**_**2**_	**+++**	**–**	**–**	short term

One pragmatic treatment regime for the interruption of agitation and psychotic symptoms is to start with higher doses of haloperidol (e.g., up to 5 mg per day in two or three doses) and then to taper the dose quickly in order to reduce the risk of extrapyramidal side effects (EPMS). If milder symptoms persist, atypical neuroleptics such as quetiapine or risperidone can be started simultanoeously at an increasing dose to maintain an antidelirogenic effect.

Physicians should be aware of potential risks and side effects of neuroleptics such as higher mortality (due to an increased rate of cardiovascular events), risk of EPMS and a higher rate of falls.

In patients with Parkinson’s disease quetiapine or clozapine should be used (as first line) since other neuroleptics bear a higher risk of worsening extrapyramidal symptoms.

Benzodiazepines have only proven to be beneficial in combination with haloperidol but not in monotherapy. The use of benzodiazepines in delirium is debated controversially given their inherent delirogenic potential. Especially in elderly patients, benzodiazepines have a significantly higher risk of side effects and should be avoided whenever possible. Therefore, we suggest that benzodiazepines should only be chosen if anxiety or extreme agitation dominate the clinical picture or in alcohol withdrawal delirium (see below). It is important to taper benzodiazepine medication as soon as possible.

In the ICU-setting, the alpha2-agonist dexmedetomidine has the most promising results in the treatment of delirium by shortening its duration and by shortening the duration of mechanical ventilation as well as the length of stay in the ICU (Table 2, Fig. 2 [10, 11, 14];). As an alternative, the alpha2-agonist clonidine may be used.

A number of studies with small sample sizes suggests beneficial effects of melatonin and melatonin receptor agonists for the prevention and treatment of delirium [10], which is currently being tested in larger randomized controlled trials.

There is no conclusive data supporting the use of medication for the prevention of delirium.

### Alcohol withdrawal delirium

For the treatment of alcohol withdrawal syndrome and alcohol withdrawal delirium, guidelines of national associations for psychiatry and neurology [15] recommend the use of benzodiazepines, clomethiazol, haloperidol, and clonidine. Adjunctive therapy with valproate, carbamazepine or levetiracetam reduces the risk of withdrawal-related epileptic seizures. Valproate has been shown to reduce the duration of pharmacological treatment and the length of hospital stay [16]. However, hepatotoxic effects have to be considered especially in patients with alcohol-induced hepatopathy.

## Conclusions

In order to prevent delirium, risk factors should be identified and taken into account when planning the hospital stay and treatment. Prevention and treatment of delirium should be based on the elimination of potential causes and on non-pharmacological interventions. Pharmacological treatment should be used in a time-limited manner and in the lowest possible dose for the management of highly stressful symptoms or high-risk behaviour if non-pharmacological treatment is not effective. The SOP provides a pragmatic algorithm for the non-pharmacological and pharmacological treatment of delirium. More randomized controlled trials are needed to improve evidence for the best treatment of delirium.

## Supplementary Information

**Additional file 1: Table S1**. Definition of delirium according to DSM-5. **Table S2.** Causes for Delirium**. Table S3.** CAM ICU**. Table S4.** ICSDC.

## Data Availability

Available to readers on request.

## Abbreviations

BPS: behavioral pain scale
CT: computed tomography
DSM-5: Diagnostic and Statistical Manual of Mental Disorders, 5th edition
EPMS: extrapyramidal motor symptoms
FORTA: Fit for the Aged list
CAM-ICU: Confusion Assessment Method in the Intensive Care Unit
ICDSC: Intensive Care Delirium Screening Checklist
ICU: intensive care unit
MRI: magnetic resonance imaging
NRS: numeric rating scale
SOP: standard operating procedure
VAS: visual analogue scale

## References

[1] Thom RP (2019). Delirium. The American Journal of Psychiatry.

[2] Wu YC (2019). Association of Delirium Response and Safety of pharmacological interventions for the management and prevention of delirium: A network meta-analysis. JAMA Psychiatry.

[3] Hui D (2017). Effect of Lorazepam with haloperidol vs haloperidol alone on agitated delirium in patients with advanced Cancer receiving palliative care: A randomized clinical trial. JAMA.

[4] 2013. *Diagnostic and statistical manual of mental disorders (5th ed.)*, Arlington, VA: American Psychiatric Association.

[5] Bergeron N (2001). Intensive care delirium screening checklist: Evaluation of a new screening tool. Intensive Care Medicine.

[6] Holt S, Schmiedl S, Thurmann PA (2010). Potentially inappropriate medications in the elderly: The PRISCUS list. Deutsches Ärzteblatt International.

[7] Wehling M (2016). VALFORTA: A randomised trial to validate the FORTA (fit fOR the aged) classification. Age and Ageing.

[8] Hshieh TT (2015). Effectiveness of multicomponent nonpharmacological delirium interventions: A meta-analysis. JAMA Internal Medicine.

[9] Pun BT (2019). Caring for critically ill patients with the ABCDEF bundle: Results of the ICU liberation collaborative in over 15,000 adults. Critical Care Medicine.

[10] *S3-Leitlinie: Analgesie, Sedierung und Delirmanagement in der Intensivmedizin (DAS-Leitlinie* 2015*)*. 2015: Deutsche Gesellschaft für Anästhesiologie und Intensivmedizin (DGAI); Deutsche Interdisziplinäre Vereinigung für Intensiv- und Notfallmedizin (DIVI).

[11] Devlin JW (2018). Clinical practice guidelines for the prevention and Management of Pain, agitation/sedation, delirium, immobility, and sleep disruption in adult patients in the ICU. Critical Care Medicine.

[12] Burry L (2018). Antipsychotics for treatment of delirium in hospitalised non-ICU patients. Cochrane Database of Systematic Reviews.

[13] Girard TD (2018). Haloperidol and Ziprasidone for treatment of delirium in critical illness. The New England Journal of Medicine.

[14] Burry L (2019). Pharmacological interventions for the treatment of delirium in critically ill adults. Cochrane Database of Systematic Reviews.

[15] *S3-Leitlinie Screening, Diagnose und Behandlung alkoholbezogener Störungen*. 2016: Arbeitsgemeinschaft der Wissenschaftlichen Medizinischen Fachgesellschaften (AWMF), Deutsche Gesellschaft für Psychiatrie und Psychotherapie, Psychosomatik und Nervenheilkunde (DGPPN), Deutsche Gesellschaft für Suchtforschung und Suchttherapie e.V. (DG-SUCHT).

[16] Farrokh, S., et al. (2020). Alcohol withdrawal syndrome in Neurocritical care unit: Assessment and treatment challenges. *Neurocritical Care*. Online ahead of print. 10.1007/s12028-020-01061-8 32794143

