# Supplementary material for: SOP: treatment of delirium
Source: Neurol Res Pract. 2021 Mar 4;3:12. doi: 10.1186/s42466-021-00110-7 (PMC7934272; doi:10.1186/s42466-021-00110-7)
Supplement: Supplementary file 1 — Additional file 1: Table S1. Definition of delirium according to DSM-5. Table S2. Causes for Delirium. Table S3. CAM ICU. Table S4. ICSDC. [file 42466_2021_110_MOESM1_ESM.docx]

SOP: Treatment of Delirium

J. Kukolja, J. Kuhn

Supplementary Material

| **Table S1**  **Definition of delirium according to DSM-5** |
| --- |
| A. Disturbance in attention (i.e., reduced ability to direct, focus, sustain, and shift attention) and awareness (reduced orientation to the environment). |
| B. The disturbance develops over a short period of time (usually hours to a few days), represents an acute change from baseline attention and awareness, and tends to fluctuate in severity during the course of a day |
| C. An additional disturbance in cognition (e.g.memory deficit, disorientation, language, visuospatial ability, or perception). |
| D. The disturbances in Criteria A and C are not better explained by a pre-existing, established or evolving neurocognitive disorder and do not occur in the context of a severely reduced level of arousal such as coma. |
| E. There is evidence from the history, physical examination or laboratory findings that the disturbance is a direct physiological consequence of another medical condition, substance intoxication or withdrawal (i.e. due to a drug of abuse or to a medication), or exposure to a toxin, or is due to multiple etiologies. |

| Table S2 Causes for Delirium | |
| --- | --- |
| Structural lesions | - Cerebral ischaemia or haemorrhage - Head trauma - Brain tumors or metastases - Brain abscesses |
| Infections | - Meningitis or encephalitis - Pneumonia - Urinary tract infections - Sepsis |
| Metabolic disorders | - Disorders of hydration or electrolyte homeostasis (sodium, potassium, calcium) - Hypoglycaemia - Hepatic or renal insufficiency - Hypovitaminosis (Vitamin B_1_ or B_12_) - Endocrine dysregulation |
| Circulation | - Circulatory shock - Anaemia - Hypoxia - Cardiac insufficiency |
| Toxic causes | - Intoxication with alcohol, cannabinoids, heroin, LSD etc. - Gas inhalation (CO, CO_2_) - Alcohol, benzodiazepine or opioid withdrawal |
| Medication (examples) | - Anticholinergic drugs - Narcotics - Benzodiazepines - Antihistaminic drugs - Corticosteroids - Drugs for Parkinson’s disease - Antiepileptic drugs - Antibiotics - Theophylline - Digoxin, Digitoxin |
| Trauma and surgery | - Severe trauma or burns - Surgery, orthopaedic or heart |
| Other | - Epilepsy, postictal state - autoimmune encephalitis - Change of environment |

| Table S3 **CAM ICU** |  |  |
| --- | --- | --- |
| **FEATURE 1: Alteration/Fluctuation in Mental Status** | Criteria | Present |
| - Is the patient’s mental status different than his/her baseline?   OR   - Has the patient had any fluctuation in mental status in the past 24 hours as evidenced by fluctuation on a sedation scale (eg, RASS, Glasgow Coma Scale [GCS]), or previous delirium assessment? | If Yes for either question | □ |
| **FEATURE 2: Inattention 1: Alteration/Fluctuation in Mental Status** |  |  |
| \| **Letters Attention Test:**  Tell the patient *“I am going to read to you a series of 10 letters. Whenever you hear the letter ‘A,’ squeeze my hand.”*  **S A V E A H A A R T**  Count errors (each time patient fails to squeeze on the letter “A” and squeezes on a letter other than “A”). \| \| --- \| | If number of errors >2 | □ |
| **FEATURE 3: Altered Level of Consciousness (LOC)** |  |  |
| - Present if the RASS score is anything other than Alert and Calm (zero)   **OR**   - If SAS is anything other than Calm (4) | If RASS $\neq$0 OR  SAS $\neq$4 | □ |
| **FEATURE 4: Disorganized Thinking** |  |  |
| **Yes/No Questions:** Ask the patient to respond:  1. Will a stone float on water?  2. Are there fish in the sea?  3. Does 1 pound weigh more than 2 pounds?  4. Can you use a hammer to pound a nail?  *Count errors (each time patient answers incorrectly).*  **Commands:** Ask the patient to follow your instructions:  a) *“Hold up this many fingers.”* (Hold 2 fingers in front of the patient.)  b) *“Now do the same thing with the other hand.”* (Do **not** demonstrate the number of fingers this time.)   If unable to move both arms, for part “b” of command ask patient to “Hold up one more finger.”  *Count errors if patient is unable to complete the entire command.* | If combined number of errors >1 | □ |
| If Features 1 and 2 are both present and either Features 3 or 4 are present: CAM-ICU is positive, delirium is present | **Delirium present** □  **Delirium absent** □ | |

| Table S4 **ICSDC** |  |  |
| --- | --- | --- |
| 1. **Altered Level of Consciousness** | **No** | **Yes** |
| - Deep sedation/coma over entire shift [SAS= 1, 2; RASS = -4,-5]   = Not assessable   - Agitation [SAS = 5, 6, or 7; RASS= 1-4] at any point   = 1 point   - Normal wakefulness [SAS = 4; RASS = 0] over the entire shift   = 0 points   - Light sedation [SAS = 3; RASS= -1, -2, -3]:   = 1 point (if no recent sedatives)  = 0 points (if recent sedatives) | **0** | **1** |
| 1. **Inattention** |  |  |
| - Difficulty following instructions or conversation - patient easily distracted by external stimuli. - Will not reliably squeeze hands to spoken letter A: **S A V E A H A A R T** | **0** | **1** |
| 1. **Disorientation** |  |  |
| - In addition to name, place, and date, does the patient recognize ICU caregivers? - Does patient know what kind of place they are in? - (list examples: dentist’s office, home, work, hospital) | **0** | **1** |
| 1. **Hallucination, delusion, or psychosis** |  |  |
| - Ask the patient if they are having hallucinations or delusions.   (e.g. trying to catch an object that isn’t there).   - Are they afraid of the people or things around them? | **0** | **1** |
| 1. **Psychomotor agitation or retardation** |  |  |
| - Either: a) Hyperactivity requiring the use of sedative drugs or restraints in order to control potentially dangerous behavior (e.g. pulling IV lines out or hitting staff) - OR b) Hypoactive or clinically noticeable psychomotor slowing or retardation | **0** | **1** |
| 1. **Inappropriate speech or mood** |  |  |
| Patient displays:   - inappropriate emotion - disorganized or incoherent speech - sexual or inappropriate interactions - is either apathetic or overly demanding | **0** | **1** |
| 1. **Sleep-wake cycle disturbance** |  |  |
| - Either: frequent awakening/< 4 hours sleep at night - OR sleeping during much of the day | **0** | **1** |
| 1. **Symptom Fluctuation** |  |  |
| - Fluctuation of any of the above symptoms over a 24 hr period. | **0** | **1** |
| **Total Shift Score:**  **Score Classification**  0 Normal  1-3 Subsyndromal Delirium  4-8 Delirium | (0-8) | |
